# Classification of Dog Breeds Using Convolutional Neural Network Models and Support Vector Machine

**DOI:** 10.3390/bioengineering11111157

**Published:** 2024-11-17

**Authors:** Ying Cui, Bixia Tang, Gangao Wu, Lun Li, Xin Zhang, Zhenglin Du, Wenming Zhao

**Affiliations:** 1 China National Center for Bioinformation, Beijing 100101, China; cuiying2019m@big.ac.cn (Y.C.); tangbx@big.ac.cn (B.T.); wugangao@gmail.com (G.W.); lil@big.ac.cn (L.L.); zhangxin@big.ac.cn (X.Z.); duzhl@big.ac.cn (Z.D.); 2 Beijing Institute of Genomics, Chinese Academy of Sciences, Beijing 100864, China; 3 University of Chinese Academy of Sciences, Beijing 100864, China

**Keywords:** dog breed classification, convolutional neural network, support vector machine, multi-network integration, feature selection, Stanford dog dataset

## Abstract

When classifying breeds of dogs, the accuracy of classification significantly affects breed identification and dog research. Using images to classify dog breeds can improve classification efficiency; however, it is increasingly challenging due to the diversities and similarities among dog breeds. Traditional image classification methods primarily rely on extracting simple geometric features, while current convolutional neural networks (CNNs) are capable of learning high-level semantic features. However, the diversity of dog breeds continues to pose a challenge to classification accuracy. To address this, we developed a model that integrates multiple CNNs with a machine learning method, significantly improving the accuracy of dog images classification. We used the Stanford Dog Dataset, combined image features from four CNN models, filtered the features using principal component analysis (PCA) and gray wolf optimization algorithm (GWO), and then classified the features with support vector machine (SVM). The classification accuracy rate reached 95.24% for 120 breeds and 99.34% for 76 selected breeds, respectively, demonstrating a significant improvement over existing methods using the same Stanford Dog Dataset. It is expected that our proposed method will further serve as a fundamental framework for the accurate classification of a wider range of species.

## 1. Introduction

Dogs were one of the earliest domestic animals and have a diversity of phenotypes. Currently, there are more than 400 dog breeds worldwide [1], and 283 breeds of them have been registered with the AKC (American Kennel Club; https://www.akc.org (accessed on 22 October 2024)). An accurate classification of dog breeds is crucial in various fields, including veterinary diagnosis, genetic disease research and pet care [2]. However, it is becoming increasingly difficult due to the diversities and similarities among dog breeds [3,4], which rely heavily on expert experiences. Therefore, identifying dog breeds easily, accurately and cost-effectively is a fascinating challenge for dog breeders, managers or fanciers.

To address the challenge of dog image identification, several methods have been proposed, which can be categorized into three main groups.

The first group consists of machine learning methods that focus on geometric features. These methods primarily involve training on geometric features extracted from dog face images and classifying these features using machine learning techniques such as principal component analysis (PCA) [5,6,7]. However, some of these methods were tested on a limited number of dog breeds (35 breeds) [5,7], while another used a larger number of dog breeds but achieved insufficient accuracy (67% for 133 breeds) [6]. PCA is a dimensionality reduction technique that aims to highlight patterns in data by emphasizing variance and capturing strong patterns in high-dimensional data. However, PCA’s effectiveness is predicated on the assumption that the data can be embedded in a globally linear or approximately linear low-dimensional space. Moreover, PCA focuses on the total variance in the explanatory variables, which does not fully reflect the amount of information, and the classification information in the original data is not fully utilized. The compressed data may even be detrimental to pattern classification.

The second group is based on convolutional neural networks (CNN) [8,9,10,11,12,13], which typically employ a single CNN model for the Stanford Dog Dataset, with most achieving an accuracy rate up to 80%. For instance, VGGNet increases network depth by using multiple 3 × 3 convolutional filters while reducing model parameters. The NIN model combines MLP and convolution, using more complex micro neural network structures in place of traditional convolutional layers. These models extract high-level semantic information to improve classification performance but may overlook contextual information around convolution and pooling kernels, leading to the loss of some feature information.

The third group combines CNN with machine learning, where several studies focusing on improving CNN models [14,15,16,17] have delivered better accuracy rates (up to 90%) than single CNN models. However, the classification accuracy is still hindered by the diversity of dog breeds. When applied to birds, cats and sheep, these models have achieved classification rates of 95% [18,19], 80% [20,21,22] and 85% [23,24,25], respectively. Notably, when using the combination of a CNN and Support Vector Machine (SVM) for flower identification, a satisfactory accuracy rate of 97% [26,27] was achieved. However, whether the accuracy of classification can be improved in dog image classification remains to be further explored.

To address this issue, we initiated this study to comprehensively research the integration of multiple CNNs and machine learning methods, with the aim of improving dog image classification accuracy. Additionally, the model was combined with dimension reducing and feature selection processing to optimize the exaction and fusion of image features. The experimental results demonstrate that our proposed model outperforms existing methods to achieve better identification efficiency.

The key contributions of this paper are given below: We propose a new method to combine CNN models and a machine learning model for dog image classification; we increased the accuracy of dog image classification to over 95%; and we used transfer learning for the CNN model training process which improved the efficiency and accuracy of this task. The rest of this paper is structured as follows: Section 2 introduces the materials and methods, including data sources, data preprocessing and model architecture; Section 3 introduce the experimental setup including parameter details; Section 4 analyzes the experimental results and discusses the selection of model combination; and finally, Section 5 discusses the findings and limitations of the study, concludes the research and explores its significance and potential applications in related fields.

## 2. Materials and Methods

### 2.1. Data Acquisition and Preprocessing

**Figure 1:**
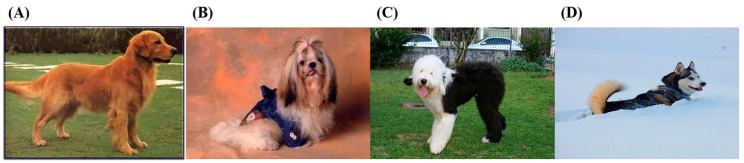
Sample images of Stanford Dog Dataset. (**A**) Golden retriever; (**B**) Shih-Tzu; (**C**) Old English sheepdog; (**D**) American Eskimo dog.

We used the Stanford Dogs Dataset [28] to train and evaluate our model, which is named DataSet1 for convenience. DataSet1 includes 20,580 annotated dog images from 120 breeds, and each breed has about 180 images. Sample images are shown in Figure 1.

**Figure 2:**
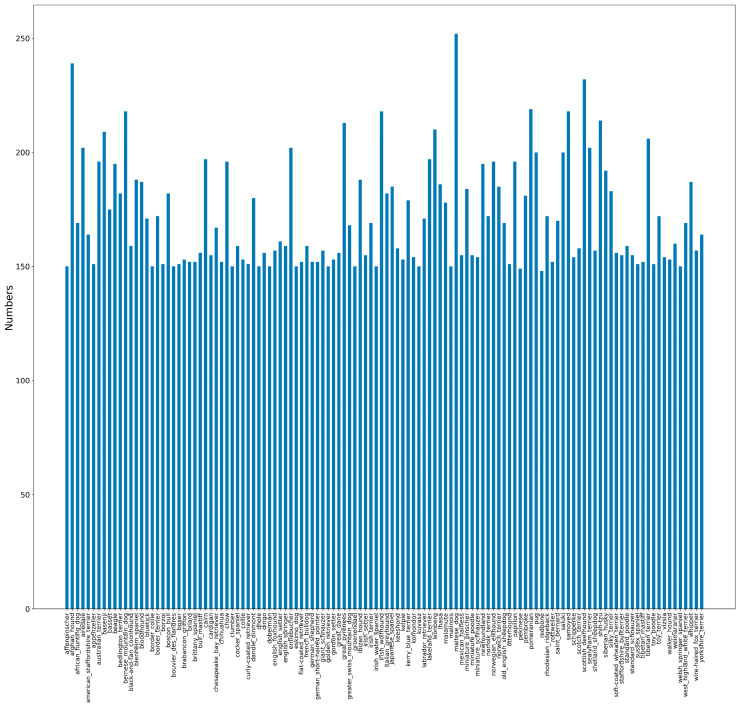
The distribution of images of each breed for Stanford Dog Dataset.

The distribution of the number of dogs for each breed is shown in Figure 2. Among these 120 breeds, Redbone has the lowest number of images with 148 and Maltese has the largest number of images with 252. To mitigate the effects of uneven data distribution on accuracy, 120 images were manually filtered for each breed according to image size, aspect ratio and background ratio to form a new dataset named DataSet2. In addition, preprocessing operations were performed on the images, including image size resetting, center cropping, and normalization. The reset image size was set according to requirements of different networks. Both datasets are divided into training and testing sets according to the ratio of 8:2 and trained separately.

### 2.2. Proposed Architecture Details

**Figure 3:**
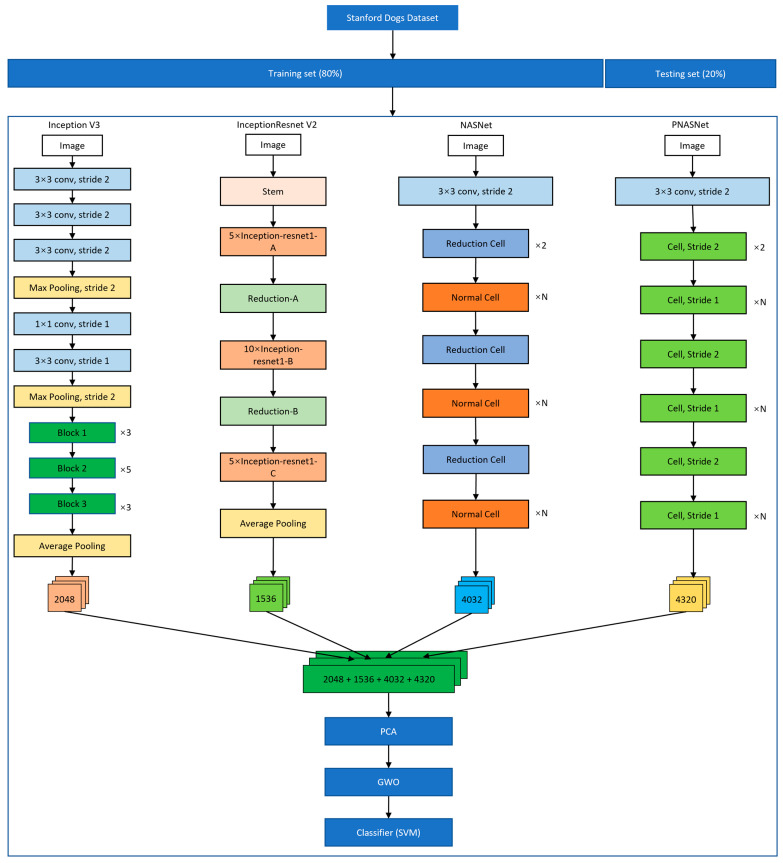
The architecture of proposed model. Dataset1 and Dataset2 are divided into an 8:2 ratio separately, 80% of the data are used for training the model and 20% is used for testing its performance. Four CNN models including Inception V3, InceptionResNet V2, NASNet and PNASNet are fine-tuned using transfer learning approach and the features before fully connected layer are extracted, respectively. The numbers 2048, 1536, 4032 and 4320 represent the number of extracted features before fully connected layer of Inception V3, InceptionResNet V2, NASNet and PNASNet, respectively, and then these features are concatenated and flattened. PCA is used to reduce the feature size and GWO is used to select the specific features. Finally, SVM is used to perform the classification task.

The model we propose is shown in Figure 3, which consists of three main steps as follows.

#### 2.2.1. Feature Extraction Using Four Known CNN Models

We used Inception V3, InceptionResNet V2, NASNet and PNASNet [29,30,31,32,33], which are independent fine-tuning models and have a high performance in ImageNet competitions to extract features before fully connected layers, respectively. Inception V3 is a part of GoogLeNet, which is a deep neural network model based on the Inception module launched by Google. Inception is used to assemble multiple convolution or pooling operations together into a network module [30]. InceptionResNet V2 is an improvement over Inception V3 by introducing residual connections, which reduces computational costs and speeds up network training [31]. NASNet is a new architecture proposed by Google in 2018, which combines features learned in ImageNet classification with the Faster-RCNN framework to exceed the best predictive performance of previously released COCO object detection task. The mean accuracy rate (mAP) of the model was 43.1%, which has 4% improvement over the best results published [33]. PNASNet is an improved model based on NASNet and proposes a search strategy using Sequential model-based optimization (SMBO). Compared with NASNet, PNASNet is 5 times more efficient, and significantly reduces the requirements of computation resources [32]. The size of each input image in this research was set to 299 × 299 pixels for Inception V3 and InceptionResNet V2, and 331 × 331 pixels for NASNet and PNASNet.

#### 2.2.2. Feature Fusion and Feature Selection Methods

The features of the above four models extracted were combined together, and then filtered using feature selection methods including principal component analysis (PCA) and the gray wolf optimization (GWO) algorithm [34] to obtain specific features. Feature selection can help to improve the accuracy of classification [35]. PCA is a linear dimensionality reduction method, which can reduce the storage space required and improve the transmission efficiency. We achieved PCA using Python’s sklearn.decomposition module. GWO is a meta-heuristic algorithm inspired by gray wolves in nature, which mimics their leadership hierarchy and hunting process. The GWO algorithm designs four agents for simulating the leadership hierarchy, which, from high to low, are gray wolf α, β, δ and ω. Here, we set the population size to 30, the number of iterations to 5 and the search range from −1 to 1, all these considered as initial parameters of the GWO algorithm, and the number of rotations was set to 30. In this study, we first used PCA and then used the GWO algorithm for feature selection.

#### 2.2.3. Classification for Dog Images

Support vector machine (SVM) was adopted to classify the above selected features, which was originally designed for binary classification problems. When dealing with multi-class problems, it is necessary to construct a suitable multi-class classifier. At present, there are two methods available: (1) The direct method, which can directly modify the objective function and combine the parameter solutions of multiple classification surfaces into an optimization problem. It is simple but difficult to implement because of its high computational complexity and is only suitable for small problems. (2) The indirect method, which mainly realizes multi-classification by combining multiple binary classifiers. Two common methods are supported, one-against-one and one-against-all [36]. In this study, we used the one-against-one strategy, which was implemented using Python’s sklearn.svm module, and we selected the RBF kernel function. In our experiments, the default parameters of the SVM were used without any hyperparameter tuning to ensure fair and reproducible results. The default parameters can perform consistently on multiple benchmark datasets and can also provide a reasonable baseline performance to focus on other aspects of the algorithm.

## 3. Experimental Setup

### 3.1. Training and Implementation Details

We integrated the three processes mentioned earlier to develop our proposed model, successfully combining CNNs with machine learning techniques. The code is implemented in Anaconda Python 3.7, and the deep learning framework is PyTorch (https://pytorch.org/ (accessed on 16 November 2024)). The whole leaning procedure is runnable on a Linux machine which is equipped with an Intel Xeon CPU with 24 GB memory and another Linux machine equipped with an NVIDIA GV100GL GPU with 32 GB memory.

All CNN models were trained using the transfer learning [37] approach in this study. The size of each input image was set to 299 × 299 pixels for Inception V3 and InceptionResNet V2, and 331 × 331 pixels for NASNet and PNASNet. These four proposed CNN models for dog breed classification were trained using a set of carefully selected hyperparameters. A batch size of 16 was chosen to balance computational efficiency and training stability. The initial learning rate was set to $1\times 10^{-3}$, and this was adjusted dynamically using a scheduler that reduced the rate by a factor of 0.1 upon detecting a plateau in validation performance. The Stochastic Gradient Descent (SGD) optimizer, known for its fast convergence and freedom from local optima, was utilized with the momentum of 0.9. The loss function we used is Cross Entropy for a multi-class classification task. The network was trained for 50 epochs. The epoch and batch size parameter values were chosen after our experimental results, and the momentum and learning rate parameter values were set according to reference [26,27]. The relevant parameters are shown in Table 1. After employing transfer learning, the features before fully connected layers of Inception V3, InceptionResNet V2, NASNet and PNASNet were extracted, with dimension of 2048, 1536, 4032 and 4320 respectively. These features were then used for feature fusion, selection and breed classification.

Data augmentation techniques were employed to enhance model robustness, including random rotations, flips and intensity variations; here, we used random resized crop, center crop and random flip. We normalize the input images before training. During training, the dataset from Stanford Dog Dataset (http://vision.stanford.edu/aditya86/ImageNetDogs/ (accessed on 22 October 2024)) named Dataset1 and images after manual filtering named Dataset2 were split into training and testing sets in an 80–20 ratio, respectively. Input images were normalized so that they have a mean of zero and a unit variance.

### 3.2. Performance Metric

In this study, the evaluation metric used for the analysis of experiments was accuracy. In the following equation, *TP* represents True positives, *TN* represents true-negative, *FP* means false-positive and *FN* means false-negative [27]:

(1) $$accuracy=\frac{TP+TN}{TP+TN+FP+TN}$$

## 4. Results

To enhance the model’s performance and the accuracy of the classification of the dog breeds, we first tried four known CNN models with good classification performances for dog images. Then, we tried to extract the features before the fully connected layer with the known CNN models mentioned and implemented classification with SVM, we also compared the effect of using a single model to extract the features and multiple models to extract the features and then fusing them on the classification accuracy. After feature fusion, we also used two feature selection methods, PCA and GWO. For the PCA method, the effects of feature dimension reduction to 5000, 4000, 3000 and 2000 on the results were compared.

### 4.1. Comparative Analysis of Single CNN Models

The single CNN model can achieve better accuracy, but not the best. We used four transfer-learning CNNs to perform the classification, including Inception V3, InceptionResNet V2, NASNet and PNASNet. The results show that NASNet and PNASNet have better classification accuracies than Inception V3 and InceptionResNet V2 (93.03% and 89.64% vs. 84.33% and 85.97% in DataSet1, 93.96% and 89.27% vs. 86.25% and 84.13% in DataSet2), and NASNet has the highest accuracy for both datasets. The results from the two datasets are closely aligned, with DataSet2 (the manually filtered dataset) showing minimal significant benefits over the other. Table 2 summarizes the classification accuracy of each CNN model.

### 4.2. The Evaluation of Fusion CNNs and SVM Classification

The combined multiple CNNs and SVM improved the classification accuracy compared with the single model shown above. In our study, fusing the the Inception V3, InceptionResnet V2, NASNet and PNASNet with SVM separately achieved a better effect in both datasets. Furthermore, fusing the merged four CNN models with SVM achieved a higher accuracy of 94.1% for 120 breeds in DataSet1 and the highest accuracy of 94.9% for 120 breeds in DataSet2. Meanwhile, we assembled any two of the CNNs mentioned above and fused them with SVM; for example, the PNASNet and NASNet (two higher accuracy models) with SVM has higher accuracy (94.2% in DataSet1 and 94.7% in DataSet2) than the combination of Inception V3 and InceptionResNet V2 (two lower-accuracy models, with 92.6% accuracy in DataSet1 and 93.5% in DataSet2). The results showed that a combination with an individual CNN model with a high accuracy overall has a better performance. Table 3 summarizes the classification accuracy of the different models.

### 4.3. Ablation Analysis of Our Model

We conducted a multi-round feature selection experiment using various feature sizes, such as 5000, 4000, 3000, and 2000, to assess classification accuracy. The experiment first employed PCA, followed by a combination of PCA and the GWO algorithm. PCA together with the GWO feature selection algorithm improved the accuracy on both datasets, which reached 94.3% on DataSet1 and 95.24% on DataSet2, respectively. Based on the combined four CNN modules above, we used PCA to reduce the features to 5000, 4000, 3000 and 2000, respectively, then used GWO to select features. To evaluate the effective influence of PCA or GWO, we validated the combined patterns of different strategies, for example, without PCA or GWO, and the results indicate that GWO has a greater impact on accuracy than PCA (Table 4). 

**Figure 4:**
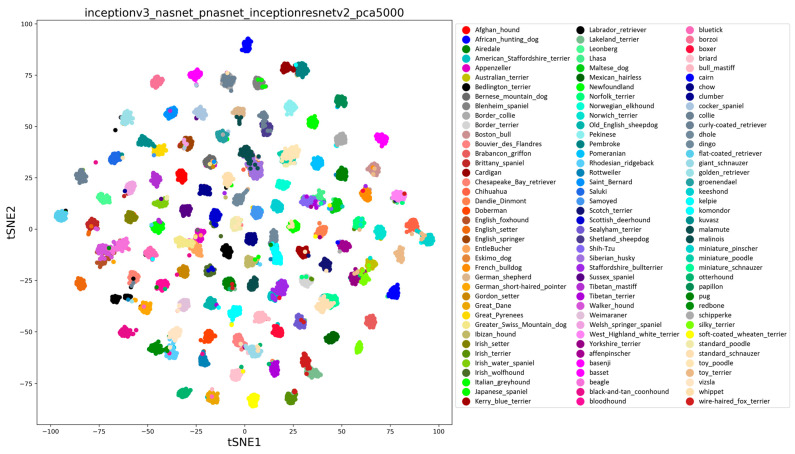
The visualized t-SNE map of extracted features from the training dataset of the 120 breeds from the Stanford Dog Dataset, which achieved the maximum classification accuracy of 95.24% on the testing dataset.

**Figure 5:**
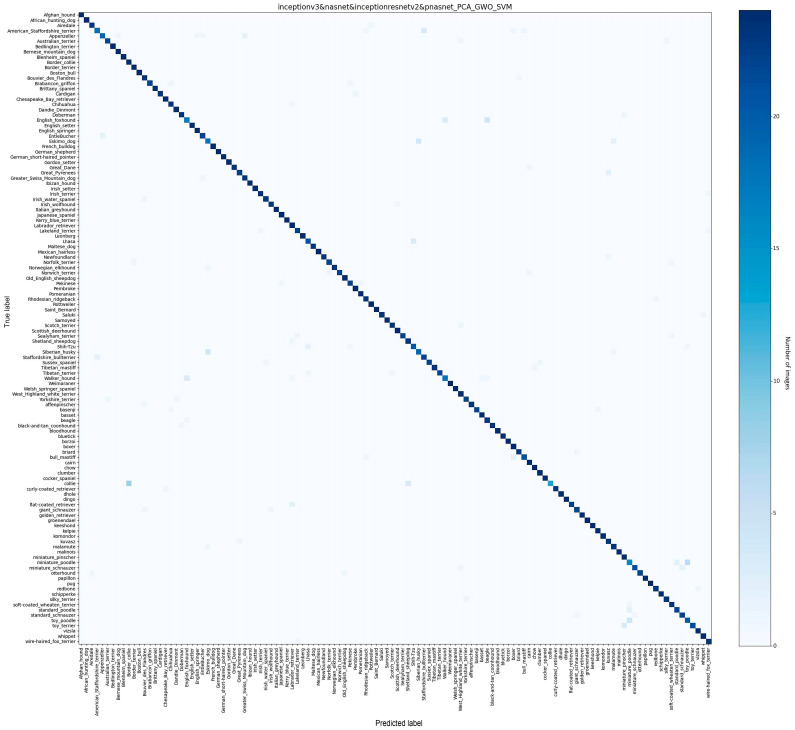
The confusion matrix of maximum classification accuracy (95.24%) for the test data for 120 breeds from the Stanford Dog Database. The y axis shows the actual labels for the dog breeds, while the x axis shows the predicted labels.

The accordingly extracted features of maximum classification accuracy (95.24%) visualized by the t-SNE method are shown in Figure 4, which clearly shows the different classifications of 120 breeds, and the corresponding confusion matrix (Figure 5) shows the same pattern.

### 4.4. Summary and Analysis of Results

In total, using the combined method mentioned above for the identification of 120 dog breeds, the average accuracy of each breed for the best model can be seen in Figure 2 and the accuracy statistics’ distribution can be seen in Table 5. There are 76 breeds with more than 95% average classification accuracy, of which 48 breeds can be distinguished perfectly with an average accuracy of 100%. Three breeds, including the English foxhound, Miniature poodle and Collie, have the lowest average accuracies, under 70%. We took a closer look at the English foxhound which has average accuracy of 67%. This breed looks similar to the Beagle and Walker hound, which are difficult to identify even by eye. In addition, the Stanford images of this breed are different in dog size, photo pose or distance. For the Miniature poodle which has average accuracy 66% and the Collie which has average accuracy 56%, the lower accuracy may be mainly caused by the vast inner differences in the breed such as coat color. We used the top 76 breeds (average accuracy above 95%) to evaluate our model, and the accuracy was up to 99.34%.

In addition, we developed an online dog image classification tool called DogVC. This tool integrates the mentioned CNN models, and was integrated into iDog [38] for NGDC [39], which is a database integrating genome, phenotype, disease and variation information of Canis lupus familiaris. DogVC is available at https://ngdc.cncb.ac.cn/dogvc/ (accessed on 22 October 2024); users can upload a dog image, and then the prediction result will be shown.

## 5. Discussion

The classification of dog breed images is a type of fine-grained image classification. The proposed model has the advantages of comprehensive feature coverage, high-quality feature output and complete automatic implementation. One of the most characteristic features of this study is that we proposed a comprehensive multi-CNNs model architecture and conducted experiments of the factors that affect accuracy including model combination, feature size and classification method. We achieved a 95.24% accuracy, which is better than other reported deep learning methods using the same Stanford Dog Dataset of 120 breeds regardless of various used hyper parameters (Table 6). Three improvements contribute to the result: Compared to the existing methods that only use a single CNN model as backbone model, we combined four CNN models and concatenated the extracted features. Our results show that the fusion of high-performance CNN models has a higher accuracy than single CNN model. (2) Existing methods directly use extracted features for classifying, while we use two feature selection methods, PCA and GWO, to obtain improved features. Our results show that PCA in combination with GWO shows better improvements in accuracy than PCA or GWO alone. (3) Most of the existing methods directly use the SoftMax function to classify dog breeds, while we use SVM. Our results show that the combination of CNN and SVM can improve accuracy. The whole set of results show the feasibility and effectiveness of our proposed model. Meanwhile, the number of images may have no direct effect on breed accuracy (Figure 2).

Historically, dog image classification has focused on dog facial geometry features such as dog face profile or facial local features such as ear shape. Due to complexity of localized features, classification tasks were only trained on limited dog breeds (35 breeds) and/or images (less than 1000 images) followed by classical machining learning methods such as PCA. Since 2012, the deep learning methods show best-in-class performance in several application tasks, which facilitate the application to dog image classification. By using the Stanford Dog Dataset, single CNN models can classify 120 breeds with state-of-the-art accuracy.

## 6. Conclusions

We started with the image data and manually filtered it based on background size and dog coat color so that there were the same number of images for each breed of dog in Dataset2; the model classification was slightly better for Dataset2 than for the raw data. The performance of multiple CNNs combined with SVM is superior to that of a single CNN combined with SVM. Two feature selection methods, PCA and GWO, also showed an improvement in dog breed classification accuracy. Selecting different CNN models for feature extraction also impacts the results.

Despite these positive outcomes from the study, there are also some limitations to consider. Firstly, the model’s accuracy may be somewhat compromised by the fact that some breeds have such subtle differences that even the human eye finds it challenging to distinguish among them, such as the English foxhound, Beagle and Walker. Secondly, the model’s accuracy is further affected by breeds with significant internal variations, like the Collie, which exhibits a diverse range of coat colors. Additionally, the complexity of the model needs to be addressed in future work.

Future research could be carried out in the following directions. Firstly, we will continue to monitor and update our proposed model in three aspects: We will incorporate the Tsinghua Dogs Dataset (https://cg.cs.tsinghua.edu.cn/ThuDogs/ (accessed on 22 October 2024)), which contains 70,428 images of 130 breeds, to increase the variety and quantity of image data, thereby enhancing the model’s generalization ability and robustness. (2) We will explore the use of modern architectures, such as autoencoders or large models in the computer vision field like Vision Transformers (ViT) [17], which can perform various vision tasks such as image classification, target detection, image segmentation, pose estimation, face recognition and so on by training on large-scale image data. (3) Extending our model to other animal images, such as cats, sheep and birds, will allow us to assess its scalability and versatility for classification tasks. Secondly, to facilitate the download and use of our model, we have developed and released all codes on BioCode (https://ngdc.cncb.ac.cn/biocode/tool/BT7319 (accessed on 22 October 2024)). And we plan to establish an email group accompanied by discussion workshops to garner feedback and suggestions. At present, our proposed model has been integrated into the iDog database (https://ngdc.cncb.ac.cn/dogvc/ (accessed on 22 October 2024)) for dog image classification, and further developing other applications such as a mobile APP will widely promote the usage of our model. And the model’s explanatory ability also be needed to improve the effectiveness of our applications. Moreover, we advocate for the collaboration of the global canine community research including researchers, veterinarians, dog owners, dog breed experts and data scientists to advance our proposed model. This would include having dog breed experts validate the image classification results using their domain knowledge, data scientists annotate and outline the images to improve classification accuracy and dog owners and veterinarians collect images to enlarge the image scale.

## Figures and Tables

**Table 1:** Parameter values of CNN architectures.

Software Used	Model	Image Size	Optimizer	Momentum	Minibatch	Learning Rate
Anaconda Python 3.7 PyTorch v1	Inception V3	299 × 299	SGD	0.9	16	$1\times 10^{-3}$
	InceptionResnet V2					
	NASNet	331 × 331				
	PNASNet					

**Table 2:** The performance of four different transfer learning CNN models.

Models	Test Accuracy (Dataset1)	Test Accuracy (Dataset2)
Inception V3	84.33%	86.25%
InceptionResNet V2	85.97%	84.13%
PNASNet	89.64%	89.27%
NASNet	93.03%	93.96%

**Table 3:** Comparative result of CNN and SVM classification.

Methods	Num of Features	Test Accuracy (Dataset1)	Test Accuracy (Dataset2)
Inception V3 + SVM	2048	89.50%	91.30%
InceptionResNet V2 + SVM	1536	92.00%	92.10%
PNASNet + SVM	4320	93.70%	94.30%
NASNet + SVM	4032	93.30%	94.50%
Inception V3 and InceptionResNet V2 + SVM	3584	92.60%	93.50%
NASNet and InceptionResNet V2 + SVM	5568	93.20%	94.60%
PNASNet and InceptionResNet V2 + SVM	5856	93.80%	94.20%
NASNet and Inception V3 + SVM	6080	93.20%	94.50%
Inception V3&PNASNet + SVM	6368	93.80%	94.20%
NASNet and PNASNet + SVM	8352	94.20%	94.70%
Inception V3 and NASNet and PNASNet and InceptionResNet V2 + SVM	11,936	**94.10%**	**94.90%**

Bold means that in both datasets, four CNNs and SVM model have better accuracy than single CNN and SVM.

**Table 4:** Ablation result of four CNN and SVM classification with feature selection.

Methods	Number of Features After PCA	Number of Features After GWO		Test Accuracy (Dataset1)	Test Accuracy (Dataset2)
		Dataset1	Dataset2		
Inception V3 and NASNet and PNASNet and InceptionResNet V2 + SVM	5000	-	-	94.00%	94.40%
	4000	-	-	94.00%	94.40%
	3000	-	-	94.00%	94.40%
	2000	-	-	94.00%	94.40%
	5000	2508	2499	94.30%	**95.24%** *
	4000	2088	2045	94.45%	95.00%
	3000	1532	1483	94.23%	95.07%
	2000	1038	1004	94.35%	94.97%
	-	5991	5974	94.28%	95.03%

* Using only PCA cannot improve the results, but after dimensionality reduction to 5000 using PCA and adding GWO algorithm, the accuracy was better in both datasets.

**Table 5:** Statistics of accuracy distribution of each breed.

Accuracy Range	Num of Breeds
1	48
0.95~0.99	28
0.90~0.95	28
0.80~0.90	10
0.50~0.75	6

**Table 6:** The results of existing studies using Stanford Dog Dataset.

Study	Year	Model/Method	Backbone CNN	Hyperparameters	Feature Selection	Classification	Accuracy
[14]	2016	Fully Convolutional Attention Networks (FCANs)	ResNet-50	Initial learning rate: 0.01, batch size: 512	no	SoftMax	88.90%
[15]	2017	Recurrent Attention Convolutional Neural Network (RA-CNN)	VGG-16	-	no	SoftMax	87.30%
[40]	2018	Fine-tuned CNN	ResNet-50	-	no	SoftMax	89.66%
[8]	2018	Fine-tuned CNN	Inception-Resnet V2	Initial learning rate: 0.1, optimizer: Nesterov, batch size: 64	no	SoftMax	90.69%
[13]	2021	Fine-tuned CNN	ResNet-50	learning rate:0.0001	no	SoftMax	90.12%
[16]	2019	Weakly Supervised Data Augmentation Network (WS-DAN)	Inception V3	Initial learning rate: 0.001, optimization: SGD, momentum: 0.9, batch size: 16	no	-	92.20%
[17]	2021	Vision Transformers (ViT)	ViT-B/16	-	no	MLP	93.20%
Proposed Approach	2022	multi-CNNs and Feature selection and SVM	Inception V3, InceptionResnet V2, NASNet and PNASNet	learning rate: 0.001, optimization: SGD, momentum: 0.9, batch size: 16	PCA and GWO	SVM	**95.24%** *

* Compared with other previous methods used on Stanford Dogs Dataset, our approach has better accuracy.

## Data Availability

The Stanford Dog Dataset can be downloaded at http://vision.stanford.edu/aditya86/ImageNetDogs/ (accessed on 22 October 2024). DataSet1 (the Stanford Dog Dataset after divided into training set and test set according to 8:2) can be downloaded at https://download.big.ac.cn/idog/dogvc/dataset_120_raw.zip (accessed on 22 October 2024). DataSet2 (the Stanford Dog Dataset after manually filtered) can be downloaded through https://download.big.ac.cn/idog/dogvc/dataset_120_10.zip (accessed on 22 October 2024). All codes can be found at https://download.big.ac.cn/idog/dogvc/code/ or https://ngdc.cncb.ac.cn/biocode/tool/BT7319 (accessed on 22 October 2024) (Here, we provide detailed usage manuals with a video). In addition, all results in this study can be available at https://download.big.ac.cn/idog/dogvc/run_result/ (accessed on 22 October 2024).

## Abbreviations

**English Abbreviations**	**Full English Title**
CNN	Convolutional Neural Network
PCA	Principal Component Analysis
GWO	Gray Wolf Optimization
SVM	Support Vector Machine
AKC	American Kennel Club
RBF	Radial Basis Function
SGD	Stochastic Gradient Descent
*TP*	True Positive
*TN*	True Negative
*FP*	False Positive
*FN*	False Negative
FCANs	Fully Convolutional Attention Networks
RA-CNN	Recurrent Attention Convolutional Neural Network
WS-DAN	Weakly Supervised Data Augmentation Network
ViT	Vision Transformers
t-SNE	t-distributed stochastic neighbor embedding
